# Update on nerve repair by biological tubulization

**DOI:** 10.1186/1749-7221-9-3

**Published:** 2014-03-07

**Authors:** Stefano Geuna, Pierluigi Tos, Paolo Titolo, Davide Ciclamini, Teresa Beningo, Bruno Battiston

**Affiliations:** 1 Neuroscience Institute of the Cavalieri Ottolenghi Foundation (NICO), University of Turin, Turin 10043, Italy; 2 Department of Clinical and Biological Sciences, University of Turin, Turin 10043, Italy; 3 Department of Traumatology, Microsurgery Unit, CTO Hospital, Turin, Italy; 4 UOC Traumatology–Reconstructive Microsurgery, Department of Orthopaedics and Traumatology, CTO Hospital, Torino, Italy

**Keywords:** Nerve reconstruction, Tissue engineering, Autotransplant, Tubulization, Vein, Skeletal muscle, Schwann cells

## Abstract

Many surgical techniques are available for bridging peripheral nerve defects. Autologous nerve grafts are the current gold standard for most clinical conditions. In selected cases, alternative types of conduits can be used. Although most efforts are today directed towards the development of artificial synthetic nerve guides, the use of non-nervous autologous tissue-based conduits (biological tubulization) can still be considered a valuable alternative to nerve autografts. In this paper we will overview the advancements in biological tubulization of nerve defects, with either mono-component or multiple-component autotransplants, with a special focus on the use of a vein segment filled with skeletal muscle fibers, a technique that has been widely investigated in our laboratory and that has already been successfully introduced in the clinical practice.

## Introduction

Nerves are complex organs that are present in almost all tissues of the human body [1] making nerve injury a very common casualty [2]. If nerve continuity is lost, surgical reconstruction is required for reconnecting nerve ends and if substance loss occurs the two stumps must be bridged [3-8]. Whereas autologous nerve grafts have been the most widely used strategy for bridging nerve gaps nonetheless this technique has disadvantages [9,10]. This observation, together with the awareness that, although possible, nerve repair and regeneration is far from being optimal [11,12], stimulated the investigation of alternative (non-nervous) conduits for repairing severe nerve defects [3,13].

In recent years, a great impulse to research in this area has been certainly represented by the significant developments in materials sciences, with the increasing availability of a number of new biomaterials and innovative manufacturing procedures [3]. However, translation to the patient of artificial synthetic nerve grafts is still limited in spite of the large body of pre-clinical research and, today, the most popular approach is still biological tubulization, i.e. the use of non-nervous autologous tissues for creating a scaffold for bridging a nerve gap. In fact, this approach is less expensive and it avoids complications due to any possible external body inflammatory reaction.

The aim of this review is to overview the most promising options for both mono-component and multiple-component autotransplants in nerve reconstruction outlining the perspectives of their translation to clinical application.

## Mono-component biological conduits

The first attempts to use non-nervous tissues for bridging a nerve defect dates back to the nineteen century and, since then, many different approaches have been tested experimentally and sometimes also with patients.

As far as our knowledge is concerned, the first attempts to bridge nerve defects with a non-nervous tissue autograft was carried out by Neuber [14], Gluck [15] and Vanlair in the second half of the 19th Century [16,17]. These authors reported the use of pieces of decalcified bone for making nerve bridges. This approach, that was soon abandoned since the results were very poor, was followed by many other attempts to create nerve guides based on the use of autologous tissues reviewed in [8,18,19].

Among the various attempts, two approaches led to particularly good results both in pre-clinical animal models and with patients, namely blood vessels and skeletal muscles and, in the following paragraphs we will focus on the use of these two types of autotransplants for nerve reconstruction.

### Blood vessels

Among the various different types of biologic tubes that have been experienced so far for bridging a peripheral nerve gaps, those based on the use of blood vessels as source of conduit material have been undoubtfully the most popular because of their large availability in the human body. Büngner, already in 1891 [20], was the first who tested a blood vessel, namely an artery segment, for repairing nerve gaps with good regeneration outcome. Although several other studies about the use of arteries as nerve guides were published in the following years [21-25], the clinical use of arteries for nerve tubulization is limited because of the difficulty in safely harvesting arteries of appropriate size in a patient. Therefore, the interest of researchers and clinicians has shifted to the use of vein segments. It was Wrede [26] in 1909^29^, the first who described the clinical use of veins for repairing nerve lesions with substance loss, reporting functional recovery after a 45-mm median nerve gap reconstruction. This report was followed by several others describing the successful use of veins as nerve guides [27-32]. The demonstration in experimental animal models that veins can lead to functional recovery comparable to autogenous nerve grafting [33,34], were followed by noteworthy clinical studies by Walton et al. [35] and Chiu et al. [36] that reported satisfactory functional recovery after sensory nerve repair by means of vein grafts, comparable to traditional autografts. The efficacy of vein grafts in bridging nerve defects in patients has been confirmed in various selected clinical conditions, such as when autologous nerve grafts are insufficient [37], for microsurgical repair of the sural nerve after nerve biopsy [38], and for repair of the inferior alveolar branch of the mandibular division of the trigeminal nerve following iatrogenic damage [39]. A recent prospective randomized study comparing polyglycolic acid and autogenous vein scaffolds for reconstruction of digital nerve gaps showed that recovery after reconstruction with a vein conduit was equivalent to polyglycolic acid conduit repair with fewer postoperative complications [40].

However, most of these studies showed that vein grafts are effective only for short nerve defects, an element that clearly represent a main limiting factor in the employment of this technique [41].

### Skeletal muscle

The idea of using skeletal muscle fibers for guiding regeneration across nerve gaps comes from the observation of the regular longitudinal alignment of muscle fibers, and especially their basal lamina, resembles the endoneurial tubes of degenerating nerves [42-44]. According to our knowledge, the first use of skeletal muscle scaffolds for nerve reconstruction was reported in 1940 by Kraus [45], followed by a series of studies which confirmed the efficacy of this approach for nerve reconstruction [44,46-51]. Up to now, only few clinical studies have been published reporting the used of muscle fiber grafts for repairing nerve defects in patients. In a series of interesting clinical papers published in the first half of the Nineties, satisfactory functional recovery was reported in most patients after reconstruction of nerve defects up to 6 cm [52-57]. In spite of these positive clinical results, the use of nerve guides made of skeletal muscle alone did not spread among surgeons so far.

## Multiple-component biological conduits

Whereas some of the mono-component biological conduits, in particular veins, have led to clinical applications, their employment is usually limited to bridging short nerve gaps and they did not gain great clinical acceptance mostly because vein-based conduits tends to collapse [41].

For this reason, various multiple-component combined biological conduits have been devised. These constructs can be based on the combination of either different tissues and organs, or the enrichment of tissue and organs with cells, trophic factors, and gene transfer.

### Multiple-tissue conduits

As regards the first option (the combination of different tissues and organs), one of the most interesting methods that have been devised is the use of vein conduits with the interposition of nerve tissue [58-60]. Although this method proved to be effective in repairing nerve defects between 2.0 cm and 4.5 cm in patients [59], it did not see a significant diffusion among clinicians.

Another interesting approach, is the use of a vein conduit filled with either fresh or pre-degenerated skeletal muscle. While the latter approach has only been addressed by few studies [61,62] because of the demonstration that pre-degeneration is not a pre-requisite for promoting nerve regeneration [63], the use of fresh skeletal muscle fibers has attracted much attention since the successful experimental validation of this paradigm by Brunelli et al. [64]. Experimental studies in laboratory animal models have shown that the fresh muscle-vein-combined guides are rapidly colonized by migratory Schwann cells (especially coming back from the distal nerve end) and that these cells maintain the capability to actively proliferate inside the conduit [65-67]. Transmission electron microscopy investigation showed that most of the grafted skeletal muscle fibers degenerate completely over the first postoperative days [64] and that new endoneurial tubes are formed very soon by perineurial cells [67].

RNA expression analysis along fresh muscle-vein combined conduits showed that degenerating skeletal muscle fibers activate an autotrophic loop based on the NRG1/ErbB system. Since the same autotrophic loop is also shared by Schwann cells, the presence of degenerating muscle tissue support Schwann cell survival and activity also by secreting NRG1 (and particularly isoform alpha) during the early nerve regeneration phases [68,69].

Quantitative assessment of both nerve fiber regeneration (using stereological methods) and functional recovery (using the grasping test) showed that the muscle-vein-combined technique lead to nerve regeneration almost comparably to autograft reconstruction, in comparison to which it avoids the secondary damage due to the healthy nerve withdrawal [13].

Yet, the muscle-vein-combined technique proved also to be effective for the simultaneous repair of two distal nerve stumps using a single proximal stump only thanks to the possibility to prepare Y-shaped conduits [70-72].

Due to its efficacy, the muscle-vein-combined nerve repair technique has already been applied in clinical case series in selected conditions such as when autograft repair was not possible [73], primary crush injuries [74] and digital nerve repair [75,76]. All clinical reports have consistently shown good clinical outcome in most cases with percentage of functional recovery similar to autologous nerve grafting. In addition, if nerve reconstruction is performed soon after lesion, the possibility of delayed autograft repair is still possible in case of failure of regeneration [74].

### Potentiation of biological conduits with cells, trophic factors or gene transfer

In alternative to the combination of different tissues/organs, some authors have attempted to enrich vein conduits with cells and/or trophic factors.

Various experimental studies showed that cell enrichment of nerve guides leads to better regeneration and recovery of the damaged nerve [77-82]. Zhang et al. [80] and Strauch et al. [78] showed that enriching a vein conduit with Schwann cells leads to successful regeneration across nerve defects as long as 40 and 60 mm (gap lengths that are not bridgeable by vein conduits alone). In the clinical viewpoint, recent progress in stem cell biology [83,84], permits to foresee that Schwann cells can even be obtained from stem cells (such as mesenchymal stem cells) harvested from the same patient [85-87].

Recently, Nijuhis et al. [82] carried out an interesting experimental study comparing repair of 15-mm sciatic nerve defect either with a vein filled with fresh skeletal muscle or with a vein filled with fresh skeletal muscle and bone marrow derived stem cells showing a tendency of the latter approach to outperforming the former one although data do not demonstrate sufficient benefit to warrant clinical implementation of stem cell enriched muscle-vein-combined conduits at this stage.

As regards trophic factor enrichment, Pu et al. [88] showed that the enriching autogenous vein grafts with nerve growth factor (NGF) improved regeneration across a 10-mm long gap of the rat sciatic nerve. The effectiveness of NGF-enriched veins as nerve conduits was confirmed by Gravannis et al. [89], using the more challenging 12-mm long gap model in the rat sciatic nerve.

Finally, also the potentiation of regeneration in the repair of the peripheral nervous system by gene transfer is getting more and more interest [90-93]. In a recent study, gene therapy has been associated with muscle-vein-combined conduits to bridge 10-mm long median nerve defects in the rat [94]. Whereas muscle-vein-combined scaffolds proved to be a good means for delivering gene therapy to regenerating nerve, results showed that particular attention should be paid in identifying the adequate gene to be transferred in order to avoid negative side effects which might even hinder the repair process and reduce functional recovery.

## Conclusions

Although regeneration potential in the peripheral nervous system is higher in comparison to the central nervous system and thus a satisfactory degree of recovery after peripheral nerve trauma can be obtained, nonetheless functional recovery is far from being optimal [1,3,11,13,95,96]. Whereas most research efforts have been directed to artificial scaffolds for bridging nerve gaps, the use of non-nervous (and thus less precious) autologous tissue as grafting material (biological tubulization) is still receiving interest from many researchers and has seen a significant spread into the clinics, in selected clinical conditions [73-76].

In particular, the use of multiple-component conduits hold promises since many experimental studies have shown that these types of nerve guides lead to a better outcome in comparison to conduits made by single components alone. Two main pieces of information arise from relevant literature. First, it has been shown that multiple-component conduits can lead to similar nerve regeneration results in comparison to traditional nerve autografts. Second, it has been shown that critical gap length limits of single-component conduits can be overcome by combined biological conduits. In particular, as regards the muscle-vein-combined technique, clinical results showed satisfactory recovery after reconstruction of nerve defects up to 4 cm in digital nerves [75] and up to 6 cm in mixed nerves of the forearm [73].

Finally, combined biological tubulization techniques, such as muscle-vein-combined nerve repair, hold also interesting perspectives in terms of tissue engineering of the peripheral nerves due to the recent demonstration that skeletal muscle fibers can be successfully infected by adeno-associated viruses (AAVs) to increase the local delivery of selected molecules (gene therapy) [94].

In conclusion, although artificial synthetic tubulization is seeing great advancements, biological tubulization still holds interesting perspectives, especially if it will be used in combination with innovative tissue engineering approaches, such as gene therapy. Although most tissue engineering approaches are still not ready for clinical translation and the risk of negative side effects must always be carefully ruled out in pre-clinical experiments [94,97], hopefully optimization of tubulization techniques would eventually provide the surgeon with an extra-amount of grafting material (with sufficient graft length too) that could be used in severe cases with multiple and large nerve substance loss.

## Competing interests

The authors declare that they have no competing interests.

## Authors’ contributions

SG wrote the first draft of the manuscript. PT, PT, DC, TB and BB integrated and completed the manuscript. All authors read and approved the final manuscript.
